# Paleomagnetic, tectonic and geochronological constraints for Permian–Triassic oroclinal bending of the Mongolian collage

**DOI:** 10.1093/nsr/nwac184

**Published:** 2022-08-31

**Authors:** Karel Schulmann, Jean Bernard Edel, Ondrej Lexa, Wenjiao Xiao, D Třebínová, Richard Spikings, Urs Schaltegger, Arkadiusz Derkowski, Marek Szczerba

**Affiliations:** Centre for Lithospheric Research, Czech Geological Survey, Czech Republic; Institut Terre et Environnement de Strasbourg, Université de Strasbourg, France; Institut Terre et Environnement de Strasbourg, Université de Strasbourg, France; Institute of Petrology and Structural Geology, Charles University, Czech Republic; Xinjiang Research Center for Mineral Resources, Xinjiang Institute of Ecology and Geography, Chinese Academy of Sciences, China; Centre for Lithospheric Research, Czech Geological Survey, Czech Republic; Earth and Environmental Sciences, Department of Earth Sciences, University of Geneva, Switzerland; Earth and Environmental Sciences, Department of Earth Sciences, University of Geneva, Switzerland; Institute of Geological Sciences, Polish Academy of Sciences, Poland; Institute of Geological Sciences, Polish Academy of Sciences, Poland

The Central Asian Orogenic Belt constitutes the Kazakhstan and Mongolian oroclinal collages located between the Tarim–North China collage (TNC) to the south and the Siberian craton to the north (Fig. 1a) [1]. While the oroclinal bending of the western Kazakhstan collage is widely accepted by the paleomagnetic and tectonic community, the oroclinal bending model of the eastern Mongolian collage is disputed [2,3]. Despite differences, all studies report: (i) difference in Late Paleozoic paleolatitudes between Siberia and the peri-Siberian terranes accreted during Cambrian–Ordovician [4,5], the Amuria and the TNC; (ii) Permian consumption of the Paleo-Asian Ocean (PAO) and (iii) Permian–Triassic counterclockwise rotations of the latter two blocks during scissor-like closure of the Mongol Okhotsk Ocean (MOO) to the north [6]. Recently acquired [2,3] and unpublished paleomagnetic data show a consistent evolution for the PAO Ordovician strata of the Mongolian Altai Wedge (MAW), the Devonian and Carboniferous oceanic rocks of the Trans-Altai Zone (TAZ), the northerly Proterozoic Lake Zone (LZ) and the Mongolian Precambrian blocks (MB) constituting the Amuria (Fig. 1a). Their joint paleomagnetic record can be grouped into five direction groups A1, A2, B, C and D, all due to successive magnetic overprinting phases (Fig. 1b). The magnetizations are carried by secondary minerals such as titanohematites for A1 and A2, titanomagnetite and magnetite for B components, and low unblocking temperatures minerals such as maghemite or sulfides for the C and D components. Coexistence of normal and reversed polarities of A1 and A2 magnetizations locate remagnetizations before or after the Kiaman reversal, i.e. Middle Carboniferous or Late Permian–Early Triassic. The second time span coincides with 270–231 Ma K–Ar ages from TAZ and MAW (Fig. 1c). Since a small part of the B components is syn-folding, the remagnetization is associated with the late Triassic–Jurassic shortening [2] while post-tectonic C and D components were acquired after the late Jurassic tightening.

**Figure 1.**
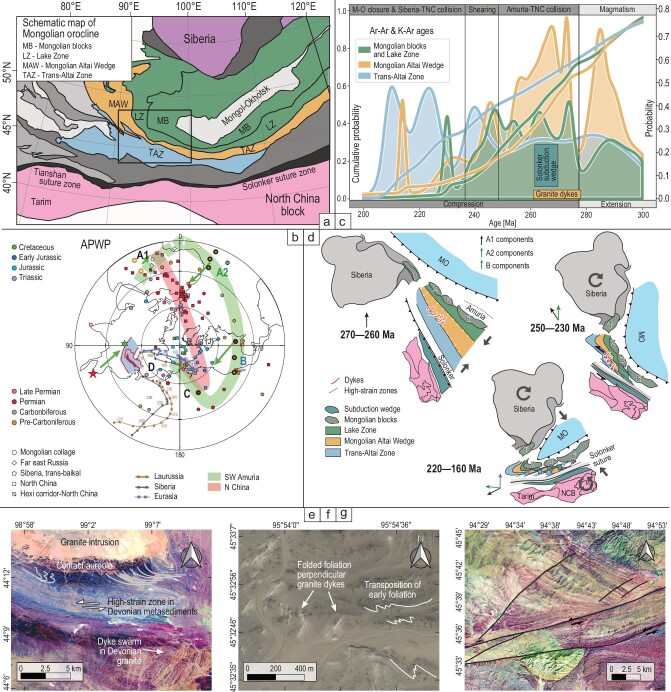
(a) Schematic map of the Mongolian part of the Central Asian Orogenic Belt with the indicated position of the tectonic zones and acquired paleomagnetic data (frame). (b) Published and new poles A1, A2, B, C and D of the Mongolian collage, the TNC and the Siberia. (c) Review of Ar–Ar and K–Ar data from the MAW, TAZ, LZ and MB. (d) Paleogeographic evolution of the LZ, MAW, TAZ and MB and the TNC derived from paleomagnetic studies. The possible reconstruction based on the A1 direction (upper-left inset) that shows distribution of Mongolian blocks Zavkhan (Z), Baydrag (B) and Erguna (E) constituting the Amuria in the north, the position of the Permian wedge of the Solonker suture in the south and deformation of the TAZ and MAW associated with intrusion of granite-pegmatite dykes (middle-left inset). The right inset shows the development of sinistral shear zones in the PAO units between rotating TNC and Amuria. The A2 direction illustrates the onset of early counterclockwise rotation. The lower-left inset shows possible reconstruction based on the B direction illustrating the final stage of the major counterclockwise rotation. The internal deformation of the southern limb of the Mongolian orocline–sinistral wrenching and pure shear shortening of the LZ, MAW and the TAZ belts (the shear corridor of Guy *et al.* [8] accommodating A1–A2 and A2–B rotations is portrayed together with the scissor-like closure of the Mongol Okhotsk Ocean and clockwise rotation of the Siberia). (e) Satellite image of the MAW with 280–260 Ma dyke swarm and S-shaped folds showing the first pure shear shortening stage. (f) 250–240 Ma sinistral shear zone in the TAZ and deformation of Permian dykes. (g) Jurassic discrete sinistral shear zone truncating 211 Ma granite in the MAW (Bugat area).

Assuming that the A1, A2 and B overprints occurred when the PAO units were tilted by ∼35° to the southwest, the corrected APWP of the MAW and TAZ overlap with the APWP of North China, implying that they have a common post-Permian evolution. The A1–A2 and the A2–B APWP reveal successive counterclockwise rotations by ≤140° (Fig. 1d). The C–D path involves a slight clockwise rotation associated with a northward drift of the TNC, PAO units and Amuria. These data can be compared with new structural and geochronological data from the MAW and TAZ which show that their Devonian to late Carboniferous N–S trending accretionary fabric was reworked by almost orthogonal E–W trending sinistral shear zones (Fig. 1e), asymmetrical ‘S-shaped’ folds (Fig. 1f) and conjugate but namely NE–SW trending sinistral shear zones (Fig. 1g) associated with E–W folding of late Permian to Jurassic successions. The collision of the TAZ and MAW was estimated by 280–260 Ma U–Pb zircon and monazite ages from a N–S trending pegmatite dyke swarm (Fig. 1e) indicating initial pure shear shortening of the PAO units [7]. These dykes and hosting units were later asymmetrically folded and transposed by sinistral shear zones forming a structural and geophysical corridor between the TNC and MB [8]. The ^40^Ar/^39^Ar and K–Ar data show three peaks at ∼270–260, 250–230 and 220–160 Ma [9–11] that are all related to: (i) mid-Permian shortening and pegmatite dyke intrusion and (ii) Triassic sinistral shearing associated with the shortening and anticlockwise rotation of pure shear dominated ‘S’-shaped lithons (Fig. 1d)lower-left inset) and development of conjugate late Triassic–Jurassic discrete shear zones [9], respectively.

The above timescales are mirrored by three-stage tectonic evolution of the Solonker suture marked by the formation or the Permian ophiolite mélange where greenschist metamorphism and deformation were dated at 269–255 Ma [12,13], Mid-Triassic NNE-directed thrusting and folding and Late Jurassic SE- and NW-directed thrusting [14]. All these data can be interpreted as a result of: (i) Permian exhumation of HP mélange along the Solonker suture associated with the pure shear 270–260 Ma shortening of the MAW and TAZ units (Fig. 1d) [11] as a result of high compressional stress transmitted from the suture to the PAO upper plate. (ii) Triassic deformation along the Solonker suture [12,13] connected with a sinistral shearing in the wide corridor comprising all the PAO units located between the Amuria and the TNC (Fig. 1d). This sinistral shearing was imposed by the counterclockwise rotation of these distinct rigid blocks resulting in progressive closure of the MOO. (iii) Jurassic massive shortening of all units located between the Siberian and TNC jaws after completion of the counterclockwise rotation (A2–B path). This scenario indicates that the counterclockwise rotation of the southern branch of the Mongolian orocline recorded by paleomagnetism can be correlated with polyphase deformation of the TAZ and MAW Paleozoic rocks, three-stage subduction-collisional evolution of the Solonker suture [13,14] and final closure of both the PAO and the MOO.

## FUNDING

This work was financially supported by the GAČR EXPRO Project 19–27682X to K.S. and O.L., the National Natural Science Foundation of China (41888101) to W.X. and the International Partnership Program of Chinese Academy of Sciences (CAS) (132744KYSB20190039). This is a contribution to IGCP 622.
